# Supplementary material for: EnzML: multi-label prediction of enzyme classes using InterPro signatures
Source: BMC Bioinformatics. 2012 Apr 25;13:61. doi: 10.1186/1471-2105-13-61 (PMC3483700; doi:10.1186/1471-2105-13-61)
Supplement: Addtional file 5 — The Java code to format the data files, evaluate and predict. The file enzml_java_code.tar.gz contains the Java code used to format database data to ARFF and XML formats, to execute cross and train-test (jackknife) evaluations and to record evaluation results to database. More information is included in the readme.txt file and the Javadoc files. The code can be used with a MySQL database. To use a different database software, other JDBC drivers might be required. [file 1471-2105-13-61-S5.gz › java_code/enzml2011/doc/uk/ac/ed/inf/enzml/mulan/learn/ResultsFormatter.html]

ResultsFormatter


---


|  |  |  |
| --- | --- | --- |
| **Overview** \| **Package** \| **Class** \| **Use** \| **Tree** \| **Deprecated** \| **Index** \| **Help** | |  |
| **PREV CLASS**   **NEXT CLASS** | **FRAMES**    **NO FRAMES**     **All Classes** |  |
| SUMMARY: NESTED \| FIELD \| CONSTR \| METHOD | DETAIL: FIELD \| CONSTR \| METHOD |  |


---


## uk.ac.ed.inf.enzml.mulan.learn Class ResultsFormatter

```
java.lang.Object
  uk.ac.ed.inf.enzml.mulan.learn.ResultsFormatter
```

---

``` public class ResultsFormatter extends java.lang.Object ```

Class

**Version:**
:   17 Aug 2010

**Author:**
:   Luna De Ferrari luna.deferrari-at-ed.ac.uk

---

**Constructor Summary**

`ResultsFormatter()`


| **Method Summary** | |
| --- | --- |
| `static uk.ac.ed.inf.utils.database.TableRow` | `addEvaluationResultsToTableRow(uk.ac.ed.inf.utils.database.TableRow row, mulan.evaluation.Evaluation results)`             adds the evaluation results to a table row object |
| `static uk.ac.ed.inf.utils.database.TableRow` | `createEvaluationRecord(int runNumber, int trainArffId, int testArffId, java.lang.String startTime, java.lang.String endTime, MulanLearner learner, mulan.evaluation.Evaluation evaluationResults, uk.ac.ed.inf.utils.database.TableManager tableManager)`             Add timestamps and identifiers to the cross evaluation results |

**Methods inherited from class java.lang.Object**

`equals, getClass, hashCode, notify, notifyAll, toString, wait, wait, wait`

**Constructor Detail**

### ResultsFormatter

```
public ResultsFormatter()
```


**Method Detail**

### addEvaluationResultsToTableRow

```
public static uk.ac.ed.inf.utils.database.TableRow addEvaluationResultsToTableRow(uk.ac.ed.inf.utils.database.TableRow row,
                                                                                  mulan.evaluation.Evaluation results)
```

:   adds the evaluation results to a table row object

---


### createEvaluationRecord

```
public static uk.ac.ed.inf.utils.database.TableRow createEvaluationRecord(int runNumber,
                                                                          int trainArffId,
                                                                          int testArffId,
                                                                          java.lang.String startTime,
                                                                          java.lang.String endTime,
                                                                          MulanLearner learner,
                                                                          mulan.evaluation.Evaluation evaluationResults,
                                                                          uk.ac.ed.inf.utils.database.TableManager tableManager)
```

:   Add timestamps and identifiers to the cross evaluation results

    :   **Parameters:**: `runNumber` -: `arffId` -: `startTime` -: `endTime` -: `learner` -: `evaluationResults` -: `tableManager` - **Returns:**: a full evaluation record


---


|  |  |  |
| --- | --- | --- |
| **Overview** \| **Package** \| **Class** \| **Use** \| **Tree** \| **Deprecated** \| **Index** \| **Help** | |  |
| **PREV CLASS**   **NEXT CLASS** | **FRAMES**    **NO FRAMES**     **All Classes** |  |
| SUMMARY: NESTED \| FIELD \| CONSTR \| METHOD | DETAIL: FIELD \| CONSTR \| METHOD |  |


---
